# Functional Capacity, Respiratory Muscle Strength, and Oxygen Consumption Predict Mortality in Patients with Cirrhosis

**DOI:** 10.1155/2016/6940374

**Published:** 2016-07-31

**Authors:** José Leonardo Faustini Pereira, Lucas Homercher Galant, Danusa Rossi, Luis Henrique Telles da Rosa, Eduardo Garcia, Ajácio Bandeira de Mello Brandão, Cláudio Augusto Marroni

**Affiliations:** ^1^Graduate Program in Hepatology, Federal University of Health Sciences of Porto Alegre, 90050-170 Porto Alegre, RS, Brazil; ^2^Graduate Program in Rehabilitation Sciences, Federal University of Health Sciences of Porto Alegre, 90050-170 Porto Alegre, RS, Brazil; ^3^Federal University of Health Sciences of Porto Alegre, 90050-170 Porto Alegre, RS, Brazil

## Abstract

*Introduction*. Liver diseases influence musculoskeletal functions and may negatively affect the exercise capacity of patients with cirrhosis.* Aim*. To test the relationship between the six-minute walk test (6MWT), maximal inspiratory pressure (MIP), and exercise capacity (VO_2peak_) measures and the survival rate of patients with cirrhosis.* Methods*. This prospective cohort study consisted of 86 patients diagnosed with cirrhosis with the following aetiology: hepatitis C virus (HCV), hepatitis B virus (HBV), and/or alcoholic cirrhosis (AC). All patients were followed up for three years and submitted to the 6MWT, pressure measurements with a compound gauge, and an exercise test (VO_2peak_).* Results*. The survival analysis showed that the individuals who covered a distance shorter than 410 m during the 6MWT had a survival rate of 55% compared with a rate of 97% for the individuals who walked more than 410 m (*p* = 0.0001). Individuals with MIPs below −70 cmH_2_O had a survival rate of 62% compared with a rate of 93% for those with MIPs above −70 cmH_2_O (*p* = 0.0001). The patients with values below 17 mL/kg had a survival rate of 55% compared with a rate of 94% for those with values above 17 mL/kg (*p* = 0.0001).* Conclusion*. The 6MWT distance, MIP, and oxygen consumption are predictors of mortality in patients with cirrhosis.

## 1. Introduction

Regardless of their aetiology, patients with cirrhosis develop a progressive worsening of the disease that can be staged using clinical and/or mathematical parameters (i.e., the Child-Turcotte-Pugh score or the Model for End-Stage Liver Disease). Disease progression is linearly correlated with morbidity and mortality [1].

In the evolved forms of the disease, patients present with markedly impaired functional capacity related to physical, cardiorespiratory, muscular, and nutritional performance that disrupts their individual and social wellbeing. These parameters may change significantly compared to those in healthy individuals and can render cirrhosis patients incapable of leading healthy lives, disrupting their lifestyles and their relationships with others [2].

Metabolic changes associated with malnutrition are a complication caused by advanced liver disease. The patient may lose a large amount of muscle mass, which leads to functional changes and physical inactivity. The combination of these factors negatively affects daily activities and quality of life in this population [3].

However, musculoskeletal and cardiorespiratory fitness have not been well studied in patients with cirrhosis. Dharancy et al. have shown that individuals with a VO_2_ below 60% of the predicted values have lower survival rates when compared to individuals with values above 60% [4]. However, in addition to the loss of muscle mass, other factors related to cardiac dysfunction associated with portopulmonary hypertension and hepatopulmonary syndrome may contribute to exercise intolerance by influencing the aerobic response and impairing the maximum oxygen uptake in patients with chronic liver disease [5].

This study aims to determine the influence of VO_2peak_, the six-minute walk test (6MWT), and maximal inspiratory pressure (MIP) measures on the survival of cirrhosis patients diagnosed with hepatitis C virus (HCV) infection, hepatitis B virus (HBV) infection, and/or alcoholic cirrhosis (AC) and followed up for three years.

## 2. Patients and Methods

This prospective cohort study was composed of a convenience sample from 86 adult patients diagnosed with HCV, HBV, and/or AC and analysed, due to the higher number of patients with these aetiologies in the institution involved and as a way of homogenization of the sample. All of the patients were part of a group of approximately 500 individuals who were monitored by the Liver Transplant Clinic of the Santa Casa de Misericordia Hospital Complex (Ambulatório de Transplante Hepático do Complexo Hospitalar Santa Casa de Misericórdia), Porto Alegre, Rio Grande do Sul, Brazil. All of the patients were also potential candidates for LTx. These patients were from December 2012 to December 2015.

In 62% of the analysed cases, cirrhosis was diagnosed via liver biopsy. The remaining patients were diagnosed via clinical and laboratory data or via endoscopic and ultrasound analysis. Patients over 65 years of age, patients with systemic or localised infection, and patients presenting with hepatocarcinoma or other associated chronic diseases were excluded from this study. All of the subjects signed a free and informed consent form and the project was approved by the Research Ethics Committee of the Hospital Complex under Opinion number 331.068.

### 2.1. Diagnostic Criteria for HCV

All of the patients underwent anti-HCV antibody analysis using the third-generation microparticle enzyme immunoassay (ELISA; AxSYM HCV version 3.0, Brazil Abbott Laboratories Ltd., Diagnostics Division). The anti-HCV-positive patients were diagnosed via qualitative HCV-RNA using reverse transcription polymerase chain reaction (RT-PCR; Amplicor, Roche Diagnostic System) with a limit of detection of 50 IU/mL, as defined by the manufacturer. These analyses were performed on two different samples with a time interval of at least three months. Patients were considered HCV-positive when they had a positive anti-HCV determination with ELISA in two readings from different samples and a positive qualitative HCV-RNA with RT-PCR. Patients with positive anti-HCV but negative qualitative HCV-RNA results were not considered HCV infected [6].

### 2.2. Diagnostic Criteria for HBV

HBV was diagnosed with ELISA using the following markers: hepatitis B surface antigen (HBsAg) and its corresponding antibody (anti-HBs) and total antibodies to the antigen for the HBV nucleocapsid (anti-HBc). The assays were performed using commercial reagents (Hepanostika, Organon Teknika BV, Boxtel, Netherlands). The quantitative detection of the anti-HBs marker was performed with serial dilutions of positive samples using concentrations that were based on the standard curve according to the manufacturer's instructions (New Hepanostika anti-HBs, Organon Teknika) [7].

### 2.3. Diagnostic Criteria for AC

The diagnosis of AC was based on a history of consuming at least 80 g of ethanol per day for a minimum of ten years [8]. All of the patients had regular medical follow-ups and were able to perform the exercise test and the 6MWT. Patients were excluded if they exhibited characteristics that would interfere with data collection, including noncooperation, haemodynamic instability, limited mobility, and neuromuscular disease.

### 2.4. Exercise Testing Protocol (VO_2peak_)

The treadmill test with gas exchange analysis was performed at Santa Casa de Porto Alegre Hospital Complex, Rio Grande do Sul, Brazil. Each patient was monitored using a three-channel Dixtal electrocardiograph coupled to an oscilloscope. Initially, the rhythm corresponding to D2 derivation was recorded for 15 minutes with the patient at rest. Subsequently, the rhythm (D2 derivation) and the electrocardiogram (derivations corresponding to V1, aVF, and CM5) were recorded simultaneously for 15 minutes with the patient under stress using the modified Bruce protocol with gas exchange analysis. Exercise was interrupted if the patient experienced symptoms that prevented continuation and/or represented a risk, including complex ventricular arrhythmias. The test was also discontinued if intraventricular and/or atrioventricular disturbances or bradyarrhythmias were observed. The tests were analysed only after the patients reached the anaerobic threshold (AT), which ensured that all patients analysed reached a maximum level of exercise during testing. AT was expressed in terms of VO_2peak_ in mL·min (STPD) and was identified by the VO_2_ value at which the respiratory exchange rate (*R* = VCO_2_/VO_2_) was equal to or greater than 1.0 and continued to increase in subsequent respiratory cycles. The arrhythmias were very important in this analysis. The total number of ventricular extrasystoles, the number of pairs of ventricular extrasystoles, and the number of sustained and nonsustained ventricular tachycardia episodes during effort and at rest were determined [9].

### 2.5. 6MWT Protocol

The 6MWT was administered to determine the patients' functional capacity, that is, the functional level of daily physical activity for each patient. This test required the patients to walk as fast as possible for the longest distance possible in six minutes. The test was conducted in a 30 m long straight and flat corridor without obstacles. Prior to taking the test, the patient was given instructions by the evaluator, who used standardised verbal encouragement at each minute of walk time and encouraged the patient to walk as far as possible. The distance was recorded at the end of the test [9]. Before and after the test, the patient was asked to report sensations of dyspnoea and lower limb fatigue using the modified Borg scale (0–10). The heart rate, respiratory rate, and peripheral oxygen saturation (SpO_2_) were also recorded at those time points.

### 2.6. Compound Gauge Protocol

To measure the strength of respiratory muscles, we used a digital compound gauge (Model 500 MVD, GlobalMed®), which was calibrated before each data collection episode. For the MIP evaluation, the participants were asked to produce maximal expiration up to a residual volume (RV). Then, the equipment was placed in the patient's mouth, and he or she was asked to produce a maximum forced inspiration. To evaluate the Muscle Expiratory Pressure, the individual was asked to initiate the procedure starting at total lung capacity, which was followed by maximal forced expiration. The procedure was conducted with the equipment properly positioned in the patient's mouth to avoid any testing failure. A nose clip was used to prevent air leakage, and the compound gauge had a pressure-release valve. The test was maintained for at least one second, and the total procedure time lasted at least two seconds, when the pressure peak was observed. The result was obtained after five procedures, with a one-minute interval between each test. At least three acceptable values with differences less than 10% were obtained. The highest pressure, measured in centimetres of water (cmH_2_O), was recorded, and the normal values recommended by the Brazilian Thoracic Society were used [10].

Statistical analysis was performed using the Statistical Package for Social Sciences (SPSS) software, Version 16.0. The Kolmogorov-Smirnov test was applied to analyse the homogeneity of the sample. The survival rate was analysed using the Kaplan-Meier method. The sensitivity and specificity of the VO_2peak_, the 6MWT, and respiratory muscle strength relative to the survival rate were determined using the ROC curve. Values with *p* < 0.05 were considered significant.

## 3. Results

The clinical and anthropometric characteristics of the sample are presented in Table 1. Sixty-six men and 20 women participated in this study. An evaluation of the three-year survival curve revealed 19 deaths (11 AC patients, five patients with HBV, and three patients with HCV). The main cause of death was decompensated cirrhosis followed by sepsis.

**Figure 1:**
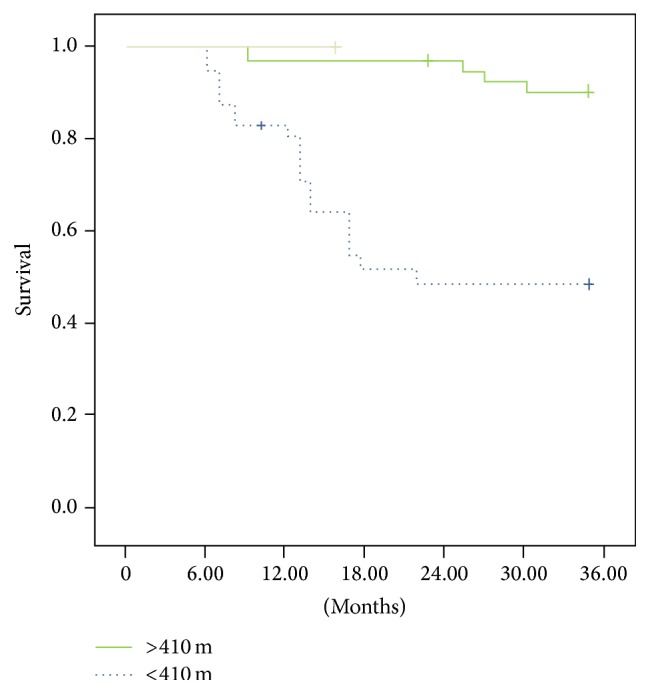
The patients who covered a 6MWT distance shorter than 410 m had a survival rate of 55% compared with a rate of 97% for those who walked more than 410 m (*p* = 0.0001). Relative risk = 4.21 (1.25–6.41, 95% CI).

The 6MWT survival analysis (Figure 1) showed that the individuals who walked a distance shorter than 410 m presented a lower survival rate (55%) compared with the 97% survival rate of those who walked more than 410 m (*p* = 0.0001; relative risk = 4.21 [1.25–6.41, 95% CI]).

**Figure 2:**
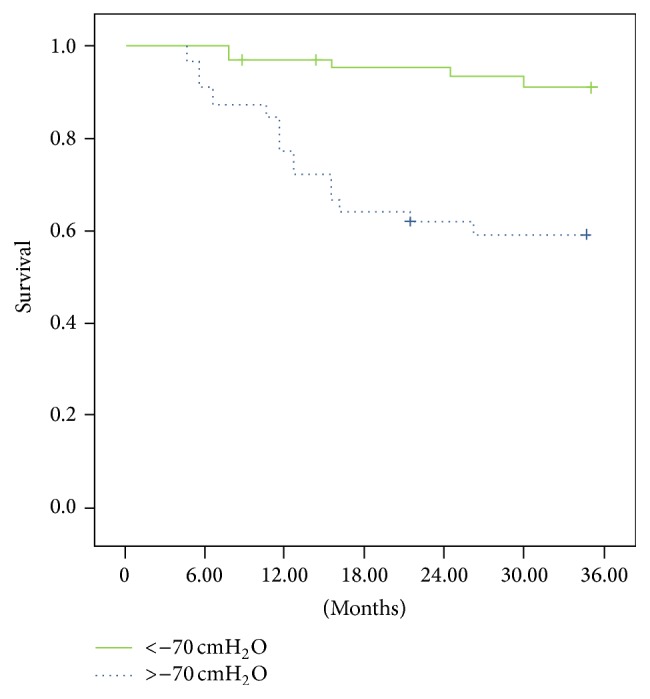
Individuals with pressure below −70 cmH_2_O had a survival rate of 62% compared with a rate of 93% for those with pressure above −70 cmH_2_O (*p* = 0.0001); relative risk = 2.25 (1.3– 5.21, 95% CI).

Regarding the MIP (Figure 2), the individuals with pressure below −70 cmH_2_O had a 62% survival rate compared with a rate of 93% for those with pressure above −70 cmH_2_O (*p* = 0.0001; relative risk = 2.25 [1.3–5.21, 95% CI]).

**Figure 3:**
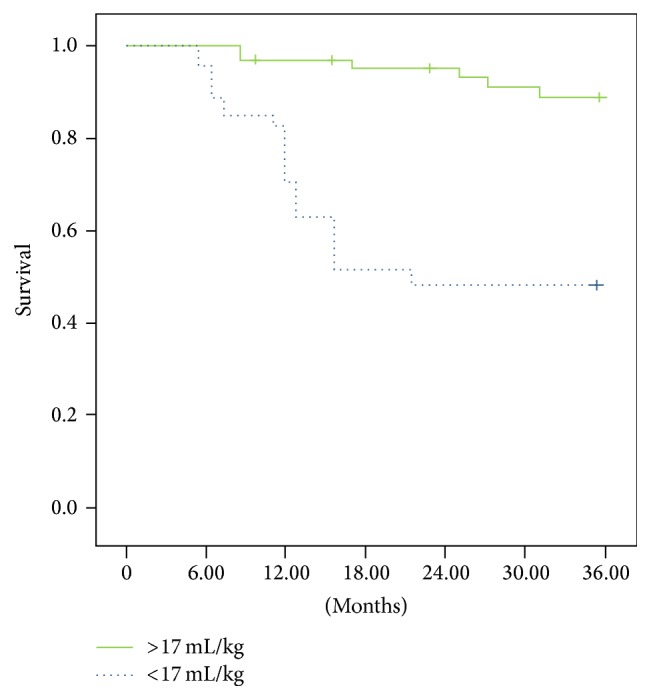
Patients with VO_2peak_ values below 17 mL/kg had a survival rate of 55% compared with a rate of 94% for patients with VO_2peak_ values above 17 mL/kg (*p* = 0.0001); relative risk = 4.10 (2.1–6.12, 95% CI).

Regarding the VO_2peak_ (Figure 3), the patients with values below 17 mL/kg had a survival rate of 55% compared with a rate of 94% for those with values above 17 mL/kg (*p* = 0.0001; relative risk = 4.10 [2.1–6.12, 95% CI]).

**Figure 4:**
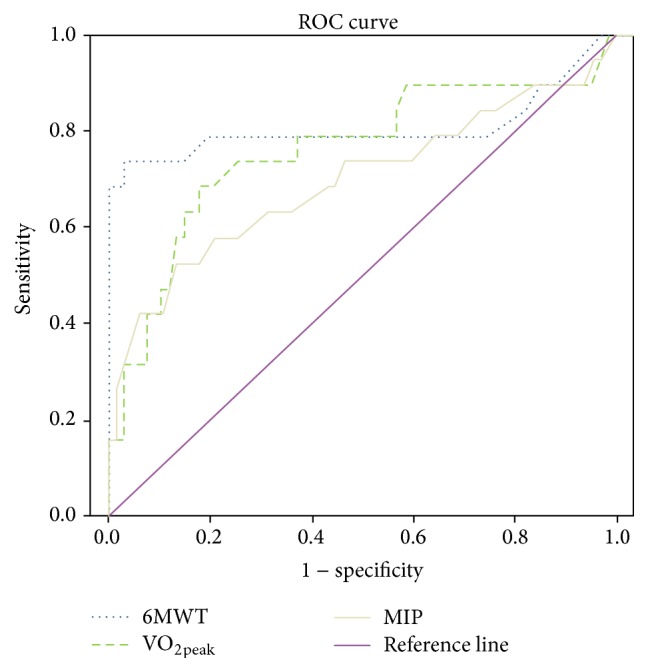
The 6MWT had an area under the ROC curve of 0.87, with a sensitivity of 92% and a specificity of 31%. The VO_2peak_ had an area under the ROC curve of 0.78, with a sensitivity of 87% and a specificity of 41%. MIP had an area under the ROC curve of 0.69, with a sensitivity of 71% and a specificity of 53%.

Regarding the ROC curve analysis relative to mortality (Figure 4), the 6MWT featured an area under the ROC curve of 0.87, a sensitivity of 92%, and a specificity of 31%. The VO_2peak_ area under the curve was 0.78, with a sensitivity of 87% and a specificity of 41%. MIP had an area under the ROC curve of 0.69, with a sensitivity of 71% and a specificity of 53%.

In the multivariate analysis (Table 2) adjusted for age, Child-Pugh score, MELD, 6MWT, VO_2peak_, MEP, and MIP were selected variables with *p* < 0.2 in the univariate Cox model. In it, the 6MWT, VO_2peak_, and MIP remain as independent predictors of mortality in the studied sample. There was a 20% higher survival rate with increasing distance in the 6MWT (HR = 0.80, CI 95% = 0.72–0.96, and *p* = 0.002), as well as a better VO_2peak_ increased by up to 30% survival (HR = 0.70, CI 95% = 0.55–0.86, and *p* = 0.001) and greater MIP increased by up to 18% patient survival (HR = 0.82, CI 95% = 0.77–0.94, and *p* = 0.002).

## 4. Discussion

This is the first study to demonstrate the influence of the VO_2peak_, 6MWT, and MIP measures on the survival rate of Brazilian patients with liver cirrhosis.

In a previous analysis conducted by our group, we described two pilot studies that correlated the severity of cirrhosis with functional capacity and respiratory muscle strength. In these studies, we demonstrated that disease severity was inversely correlated with the respective variables [11, 12].

In a recent study, patients with significantly limited functional capacity showed higher mortality compared with more physically fit patients [13], showing that the musculoskeletal dysfunction related to liver disease negatively affects patients' exercise capacity.

A possible explanation for the reduction in VO_2peak_ might be related to the loss of muscle mass in cirrhosis patients; however, the VO_2peak_ reduction may also be caused by the decrease in mitochondrial oxidative capacity and/or the number of mitochondria in the muscle tissue. The adenosine triphosphate (ATP), phosphocreatine (PCr), and total magnesium levels are reduced in the skeletal muscle of patients with cirrhosis. This change was previously described by Jacobsen et al. [14], who showed that the highest indices of mitochondrial ATP and PCr were found in individuals classified as Child-Pugh A in comparison to patients classified as B and C.

The exercise limitations that commonly affect patients with liver diseases can be observed by the decrease in VO_2peak_ during the cardiopulmonary stress test. The reduction in aerobic capacity that decreases the oxygen uptake may be a secondary result of negative chronotropism combined with cardiac output dysfunctions and the end-diastolic volume of the left ventricle, a characteristic of cirrhotic cardiomyopathy [15–18]. Hepatopulmonary syndrome also contributes to a decreased oxygen index and results in severe hypoxaemia during exercise. In addition, a reduced VO_2_ may result in a decrease in peripheral tissue perfusion, which damages the muscular system and negatively impacts the fitness of liver disease patients [19, 20].

Alameri et al. evaluated the functional status of patients using the 6MWT distance and found that patients with cirrhosis performed poorly on the test, covering only 306 m. The authors showed that mortality was correlated with the distance covered. Patients with cirrhosis and more severe Child-Pugh scores had a lower survival rate and a lower total distance covered compared with patients with HCV, HBV, and hepatitis A [21].

The impact of liver diseases can deteriorate the muscular system, which was evident from the higher mortality rate of the patients who had the lowest respiratory muscle strength values.

The ROC curve shows how the 6MWT, VO_2peak_, and MIP measures influence the survival rate. However, submaximal exercise has been shown to be the best predictor of survival in patients with cirrhosis, and functional capacity is an important variable in monitoring the survival of these patients.

The limitations observed in the 6MWT, VO_2peak_, and MIP results reflect the physical deconditioning of these patients. The development of pre- and posttransplant rehabilitation programs has been cited as an option for improving the physical limitations caused by cirrhosis [21].

While the potential malnutrition framework can influence exercise capacity, it is important to note that our patients were previously evaluated by a nutritionist through Bioelectrical Impedance Analysis. A limitation of this study was carrying out evaluations only when there is an opportunity, something that can be corrected in future works.

We have demonstrated the strong influence of functional status, oxygen consumption, and respiratory muscle strength on the survival rate of patients with cirrhosis, showing that muscular and cardiorespiratory impairment may predict mortality in these patients.

Given that the liver disease causes a long period of physical inactivity and waiting until the transplant is long, causing the appearance of more complications, the development of a more specific rehabilitation program is needed for this population, which enables reducing physical inactivity, very present in these patients, and maybe improves posttransplant results. Thus, further studies should be conducted so we can better discuss these results.

## Figures and Tables

**Table 1:** The patients' anthropometric and clinical characteristics.

Age (years)	56.3 ± 8.1
Sex (M/F)	66/20
Weight (kg)	67.34 ± 8.62
Height (m/cm)	1.67 ± 0.83
BMI	24.10 ± 1.21
Diagnosis (*n*)	
HCV	40
HBV	16
AC	30
Alb (g/dL)	4.3 ± 0.3
ALT (U/L)	21.5 ± 4.6
AST (U/L)	25.1 ± 7.1
Death (*n*)	19
MELD	17 ± 3
MIP	−70 ± 13.71
VO_2peak_	17.06
6MWT (m)	410 ± 27.84

Age, weight, height, and BMI are shown as the mean ± standard deviation. M/F = male/female. BMI = body mass index. HCV group = hepatitis C virus group. HBV group = hepatitis B virus group. AC group = alcoholic cirrhosis group. Alb = albumin. AST = aspartate aminotransferase. ALT = alanine aminotransferase. MELD = model for end-stage liver disease. VO_2peak_ = highest oxygen uptake. MIP = maximum inspiratory pressure. 6MWT = six-minute walk test. m = metres.

**Table 2:** Univariate and multivariate analysis of the variables under study.

Variable	Not adjusted analysis		Adjusted analysis	
	HR (95% CI)	*p* value	HR (95% CI)	*p* value
*Age*	1.05 (0.97–1.14)	**0.182**	1.02 (0.91–1.14)	0.742
*Gender*				
Female	1.0			
Male	0.6 (0.26–1.76)	0.426		
*Child-Pugh*				
Class A	1.0			
Class B	1.65 (0.27–9.86)	0.584	1.55 (0.19–13.7)	0.665
Class C	17.9 (3.98–80.8)	**0.01**	3.42 (0.35–35.8)	0.288
*MELD*	1.33 (1.11–1.60)	**0.02**	1.22 (0.81–1.6)	0.251
*6MWT *	0.83 (0.77–0.91)	<**0.01**	**0.80 (0.72–0.96)**	**0.002**
*VO*_*2peak*_	0.45 (0.33–0.67)	<**0.01**	**0.70 (0.55–0.86)**	**0.001**
*MIP*	0.75 (0.65–0.77)	<**0.01**	**0.82 (0.77–0.94)**	**0.002**
*MEP*	0.83 (0.73–0.91)	<**0.01**	1.06 (0.87–1.5)	0.525

MELD: model for end-stage liver disease, 6MWT: six-minute walk test,  VO_2peak_: peak oxygen consumption, MIP: maximum inspiratory pressure, and MEP: Maximum Expiratory Pressure. HR = hazard ratio; analysis adjusted by age, Child-Pugh score, MELD, 6MWT, VO_2peak_, MIP, and MEP (*p* < 0.2).

## Abbreviations

6MWT: Six-minute walk test
MIP: Maximal inspiratory pressure
VO_2peak_: Exercise capacity peak
HBV: Hepatitis B virus
HCV: Hepatitis C virus
AC: Alcoholic cirrhosis
VO_2_: Exercise capacity
HBsAg: Hepatitis B surface antigen
anti-HBs: Antibody B surface
RV: Residual volume
cmH_2_O: Centimetres of water
SPSS: Statistical Package for Social Sciences
Alb: Albumin
ALT: Alanine aminotransferase
AT: Anaerobic threshold
AST: Aspartate aminotransferase
BMI: Body mass index
M/F: Male/female
MEP: Muscle Expiratory Pressure
MELD: Model for End-Stage Liver Disease.

## References

[1] Hong S.-H., Kim J.-E., Cho M.-L. (2011). Comparison of the Child-Turcotte-Pugh classification and the model for end-stage liver disease score as predictors of the severity of the systemic inflammatory response in patients undergoing living-donor liver transplantation. *Journal of Korean Medical Science*.

[2] Foroncewicz B., Mucha K., Szparaga B. (2011). Rehabilitation and 6-minute walk test after liver transplantation. *Transplantation Proceedings*.

[3] Robinson L. R., Switala J., Tarter R. E., Nicholas J. J. (2000). Functional outcome after liver transplantation. *Archives of Physical Medicine and Rehabilitation*.

[4] Dharancy S., Lemyze M., Boleslawski E. (2008). Impact of impaired aerobic capacity on liver transplant candidates. *Transplantation*.

[5] Maganty K., Ghanta R., Bejarano P. (2011). Liver transplantation for hepatopulmonary syndrome due to noncirrhotic portal hypertension. *Transplantation Proceedings*.

[6] Hutin Y., Kitler M. E., Dore G. J. (2004). The global burden of Hepatitis C working group. Global burden of disease (GBD) for hepatitis C. *Journal of Clinical Pharmacology*.

[7] Said Z. N. A. (2011). An overview of occult hepatitis B virus infection. *World Journal of Gastroenterology*.

[8] Tilg H., Day C. P. (2007). Management strategies in alcoholic liver disease. *Nature Clinical Practice Gastroenterology & Hepatology*.

[9] American College of Sports Medicine (1995). *Guidelines for Exercise Testing and Prescription*.

[10] Sociedade Brasileira de Pneumologia e Tisiologia (2002). Diretrizes para testes de função pulmonar. *Pneumology Brazil Journal*.

[11] Galant L. H., Forgiarini L. A., Dias A. S. (2011). The aerobic capacity and muscle strength are correlated in candidates for liver transplantation. *Arquivos de Gastroenterologia*.

[12] Galant L. H., Ferrari R., Forgiarini L. A., Monteiro M. B., Marroni C. A., Dias A. S. (2010). Relationship between MELD severity score and the distance walked and respiratory muscle strength in candidates for liver transplantation. *Transplantation Proceedings*.

[13] Carey E. J., Steidley D. E., Aqel B. A. (2010). Six-minute walk distance predicts mortality in liver transplant candidates. *Liver Transplantation*.

[14] Jacobsen E. B., Hamberg O., Quistorff B., Ott P. (2001). Reduced mitochondrial adenosine triphosphate synthesis in skeletal muscle in patients with child-pugh class B and C cirrhosis. *Hepatology*.

[15] Campillo B., Fouet P., Bonnet J. C., Atlan G. (1990). Submaximal oxygen consumption in liver cirrhosis: evidence of severe functional aerobic impairment. *Journal of Hepatology*.

[16] Epstein S. K., Ciubotaru R. L., Zilberberg M. D. (1998). Analysis of impaired exercise capacity in patients with cirrhosis. *Digestive Diseases and Sciences*.

[17] Beyer N., Aadahl M., Strange B. (1999). Improved physical performance after orthotopic liver transplantation. *Liver Transplantation and Surgery*.

[18] Krasnoff J. B., Vintro A. Q., Ascher N. L. (2006). A randomized trial of exercise and dietary counseling after liver transplantation. *American Journal of Transplantation*.

[19] Epstein S. K., Zilberberg M. D., Jacoby C., Ciubotaru R. L., Kaplan L. M. (1998). Response to symptom-limited exercise in patients with the hepatopulmonary syndrome. *Chest*.

[20] Campillo B., Fouet P., Bonnet J. C., Atlan G. (1990). Submaximal oxygen consumption in liver cirrhosis. Evidence of severe functional aerobic impairment. *Journal of Hepatology*.

[21] Alameri H. F., Sanai F. M., Al Dukhayil M. (2007). Six minute walk test to assess functional capacity in chronic liver disease patients. *World Journal of Gastroenterology*.

